# Identification of immune-associated genes in vascular dementia by integrated bioinformatics and inflammatory infiltrates

**DOI:** 10.1016/j.heliyon.2024.e26304

**Published:** 2024-02-10

**Authors:** Fangchao Wu, Junling Zhang, Qian Wang, Wenxin Liu, Xinlei Zhang, Fangli Ning, Mengmeng Cui, Lei Qin, Guohua Zhao, Di Liu, Shi Lv, Yuzhen Xu

**Affiliations:** a Department of Rehabilitation Medicine, Sir Run Run Shaw Hospital, Zhejiang University School of Medicine, Hangzhou 310016, China; b Shandong Medicine Technician College, Taian 271000, China; c Department of Central Laboratory, The Affiliated Taian City Central Hospital of Qingdao University, Taian, 271000, China; d Department of Rehabilitation, The Second Affiliated Hospital of Shandong First Medical University, Taian, 271000, China; e Department of Neurology, Dongping County People's Hospital, Taian, 271000, China

**Keywords:** Immune, miRNA, Vascular dementia, Bioinformatics, Inflammatory

## Abstract

**Objective:**

Dysregulation of the immune system plays a vital role in the pathological process of vascular dementia, and this study aims to spot critical biomarkers and immune infiltrations in vascular dementia employing a bioinformatics approach.

**Methods:**

We acquired gene expression profiles from the Gene Expression Database. The gene expression data were analyzed using the bioinformatics method to identify candidate immune-related central genes for the diagnosis of vascular dementia. and the diagnostic value of nomograms and Receiver Operating Characteristic (ROC) curves were evaluated. We also examined the role of the VaD hub genes. Using the database and potential therapeutic drugs, we predicted the miRNA and lncRNA controlling the Hub genes. Immune cell infiltration was initiated to examine immune cell dysregulation in vascular dementia.

**Results:**

1321 immune genes were included in the combined immune dataset, and 2816 DEGs were examined in GSE122063. Twenty potential genes were found using differential gene analysis and co-expression network analysis. PPI network design and functional enrichment analysis were also done using the immune system as the main subject. To create the nomogram for evaluating the diagnostic value, four potential core genes were chosen by machine learning. All four putative center genes and nomograms have a solid diagnostic value (AUC ranged from 0.81 to 0.92). Their high confidence level became unquestionable by validating each of the four biomarkers using a different dataset. According to GeneMANIA and GSEA enrichment investigations, the pathophysiology of VaD is strongly related to inflammatory responses, drug reactions, and central nervous system degeneration. The data and Hub genes were used to construct a ceRNA network that includes three miRNAs, 90 lncRNA, and potential VaD therapeutics. Immune cells with varying dysregulation were also found.

**Conclusion:**

Using bioinformatic techniques, our research identified four immune-related candidate core genes (HMOX1, EBI3, CYBB, and CCR5). Our study confirms the role of these Hub genes in the onset and progression of VaD at the level of immune infiltration. It predicts potential RNA regulatory pathways control VaD progression, which may provide ideas for treating clinical disease.

## 1 Introduction

It is considered the second most typical kind of dementedness after Alzheimer's disease, accounting for 20% of all demented cases. For a multitude of reasons, long-term chronic hypoperfusion of the entire brain or specific brain regions results in vascular dementia (VaD), which eventually advances to a severe cognitive impairment syndrome [1]. Other features include behavioral abnormalities, motor abnormalities, and autonomic dysfunction [2]. There are no precise diagnostic or classification criteria for VaD, so there's no proper clinical treatment [3]. The underlying mechanisms of VaD episodes are primarily undefined and may be related to pathological processes. Wherever immunity is located in the central nervous system, neuroinflammation is an immune cascade reaction mediated by interstitial tissue cells. An immune cascade reaction called neuroinflammation is controlled by interstitial tissue cells. Various harmful occurrences, such as infection, ischemia, and trauma, also bring on the inflammatory response. Ischemia caused by chronic under-perfusion will over-activate neuroinflammation and cause pathological changes like the barrier, triggering or intensifying the onset and progression of VaD [4,5]. Significantly changed protein spots were detected by HPLC-MS/MS in one research after they were isolated using 2-D DIGE [6]. Electrophysiological experiments confirmed that the proteins that have been changed are involved in energy metabolism and cytoskeletal structure. Cerebral blood flow gradually decreases with chronic cerebral hypoperfusion, which causes cognitive decline and neurodegenerative illnesses such as vascular dementia. The synaptic proteome may reflect metabolic alterations and deficiencies in neuroplasticity brought on by decreased oxygenation and energy availability. There is a need to investigate the molecular causes of VaD and prospective diagnostic tools and treatment options.

Bioinformatics has been widely applied to spot biomarkers and therapeutic targets for diseases and the mechanisms underlying the events of various medical specialty disorders and dementedness [7]. This makes use of a variety of methods, including technology and optogenetics. One review [8] summarizes optogenetics' application in neurobiology, usually applied with adenoviruses expressing photosensitive proteins. There may be a concentration of adenoviruses in particular nerve areas. The target nerve area is exposed to radiation to regulate the adenovirus-encoded photosensitive protein. The light-controlled photosensitive protein may be activated or inhibited through selective ion transit inside and outside the cell membrane. The method has been extensively applied to treating dementia in recent years. Optogenetics may be used to research brain circuits, regulate nerve cells, and cure dementia by changing the state of neurons. Also, Machine learning has played a significant role in exploring the mechanisms of neurological diseases. According to research [9], machine learning might improve the Parkinson's prediction channel and provide new opportunities for understanding the underlying mechanics of the illness. At the patient or population level, explanations can facilitate the development of preventative medicines by identifying characteristics linked to particular phenotypes or phases. Brain imaging procedures can be influenced at other stages by identifying areas of interest for modeling. For instance, acquisition sequences can be adjusted in real-time to best image certain regions of interest. Clinical professionals who can pinpoint the main factors that led to a patient's diagnosis can make significant progress toward personalized therapy with the help of customized, patient-specific interpretation. In the future, interpretable models can aid in advancing scientific research by discovering new biomarkers.

Several bioinformatics studies on Alzheimer's and the essential genes related to AD have mostly been known [10,11]. According to studies, the system may play a significant role in the advanced pathophysiology of dementia [12]. For instance, T cells may contribute to accommodating immune responses during AD episodes by penetrating brain tissue through the anatomical structure [13]. Intrathecal compartments may be impacted by cytomegalovirus (CMV) reactivation-related alterations in peripheral T cells, even though CMV is seldom the source of CNS infections in immunocompetent hosts [14]. T cells activated in the peripheral are likely to travel to the central nervous system (CNS) because specific immune cells may penetrate the blood-brain barrier. This migration could result in neurodegeneration. In this investigation, t-cell activation was not limited to intrathecal compartments [13], and peripheral CD8 T cell activity was substantially correlated with higher numbers of active CD8 T cells in the CSF. As a result, T cell activation in AD could have happened early in the peripheral disease course, and these T cells might have crossed the blood-brain barrier (BBB). Nevertheless, the results indicate a particular immunological response in the early stages of the illness, independent of whether immune activation is causative or derived. These results might contribute to the development of activated CD8 T cells in CSF fluid as an early diagnostic biomarker. Understanding the migration of neutrophils and their potential protective or detrimental effects will be crucial for comprehending the pathophysiology of AD, particularly in connection to inflammation in the brain. According to several investigations, immune cells infiltrating the tissue aid in tissue healing by phagocytosing Aβ [[15], [16], [17]]. Furthermore, recent research shows that microglia can successfully invade neutrophils [18]. These data suggest that active neutrophils may phagocytose amyloid plaques, which are subsequently removed by microglia. Overall, research has demonstrated [19] that immune cells may interact with senile plaques in the brain to impact the course of Alzheimer's disease. A new understanding of the etiology and possible therapies for AD may result from understanding the interactions between neutrophils and senile plaques. In addition, Different forms of immune cells, monocytes, and macrophages were found to be extensively concerned with the pathological process of AD [20]. However, VaD has received very little attention.

The use of microarray analysis of biofunctional pathways, bioinformatics, and differentially expressed genes (DEGs) in the development of vascular dementia expands the reach of earlier genome-based studies of genetic alterations [21], and a cross-disciplinary approach called bioinformatics may be utilized to investigate the biological causes of illness[22]. With more accurate knowledge of the molecular networks and genes implicated in these networks, the pathophysiology and therapy of the molecular networks and genes linked to vascular dementia may be improved. Any investigations are necessary to identify new treatment targets for many trustworthy diagnostic biomarkers to overcome the discrepancies discovered in earlier studies. This work aimed to pinpoint important immune infiltration expression in VaD bioinformatics, identify essential Hub genes in VaD bioinformatics, and investigate their intrinsic mechanisms. We have created ceRNA regulatory networks and used the DSigDB database to predict therapeutic drugs that may be effective against VaD. By identifying crucial signs of aberrantly expressed genes and immune infiltration, we can better understand the molecular processes underlying vascular dementia.

## 2 Materials and methods

### 2.1 Data source and identification of associated immune differentially expressed genes

The public dataset GSE122063 (https://www.ncbi.nlm.nih.gov/geo/query/acc.cgi?acc=GSE122063), which contains brain samples from 44 healthy individuals, 36 individuals with vascular dementia, and 56 individuals with Alzheimer's (AD), was downloaded from the GEO database[23]. Only the gene expression data from the 36 VaD patients and the 44 matched controls were kept. We conduct differential analysis using the R package limma to find overlapping DEGs and obtain differential genes between the comparison team and VaD. The log2 fold change |log2FC| >1 and adj. *P* < 0.05 criteria for differential genes were fully set, indicating DEGs with increased expression. Likely downregulated DEGs are indicated by |log2FC|< −1 and adj. *P* < 0.05. Heat maps and volcano plots are used to display the outcomes.

To obtain the immune genes, we screened from the ImmPort [24], GenegCards [25], and MSigDB databases [26] and obtained 1795, 21127, and 20451 immune genes, respectively, and finally obtained 1321 immune genes by taking the intersection. Interacting DEGs and immune genes frequently detect differentially expressed immune genes.

### 2.2 Weighted Gene Co-expression network analysis

WGCNA [27] created a co-expression network in DEGs that met the requirements for a scale-free topology. We first separately calculated each gene's median absolute deviation (MAD) and removed the top 50% of genes with the least MAD to exclude outliers and samples. After investigating all DEGs using the WGCNA package in R code, the soft thresholding power was discovered. Following the creation of the weighted co-expression network, DEGs were grouped into several modules with radically varied color designations. The relationship between each module was further investigated. A crucial module for any enrichment study was believed to be the one that correlated with VaD the most.

### 2.3 PPI network construction

Using the Kegg REST API, we obtained the most current KEGG Pathway sequence annotation used as the backdrop to map the sequence into the background set (https://www.Kegg.jp/Kegg/rest/Keggapi.html) [28]. The series set enrichment findings are produced using ClusterProfiler's enrichment analysis. For the background mapping of genes for the enrichment analysis of sequence sets, we use the GO annotation of genes in the R software program. Structured information about gene products is available from the Gene Ontology (GO) framework (2019). The rigorous examination of gene activities uses an extensive database called the Kyoto Encyclopedia of Genes and Genomes (KEGG) [29]. *P* < 0.05 indicates a significant difference.

The STRING online database [30] was used to build the PPI network in the required module. The Cytoscape package system then produced an image of the PPI network [31]. The MCODE module [32] in the Cytoscape package was further applied to cluster the PPI network with the settings "degree cutoff = 2, max. depth = 100, k-core = 2, node score cutoff = 0.2." The PPI network was clustered to look for densely connected regions and identify hub genes among the PPI network.

### 2.4 Machine learning

Two machine learning algorithms were employed to examine candidate genes for VaD diagnosis. LASSO is a regression technique that may be used to choose a variable to strengthen the prediction accuracy and to select a variable and regularize it to enhance the prediction accuracy and understandability of an applied mathematics model [33]. The benefits of RF include greater accuracy, sensitivity, and specificity, as well as no constraints on changing conditions. This method can predict continuous variables and spot patterns without apparent fluctuations [34]. The LASSO regression and RF analysis were carried out using the R packages "glmnet" [35] and "randomForest" [36]. LASSO and RF intersection genes were suggested as possible hub genes for diagnosing VaD.

### 2.5 Validating the VaD-expressed genes database

GSE186798(GPL23159) included ten samples each of vascular dementia-associated post-stroke dementia, post-stroke non-dementia samples, and ten samples from healthy controls. From these, only the fit and post-stroke dementia samples were chosen. Box plots were made to determine whether the inferred VaD target genes were significantly differentially expressed in the validation set samples, showing that this was no accident.

### 2.6 Nomogram construction and ROC analysis

The structure of nomograms is effective for identifying clinical VaD. The R package "rms" was utilized to build the representation based on candidate genes [37]. the PROC package was used to assess the diagnostic predictive value of critical genes. VaD identification was shown using the area under the curve (AUC) and 95% confidence intervals (CI). AUC >0.7 was regarded as the most straightforward diagnostic cost. The online String tool created the network to examine the relationships between the recognized genes [38].

### 2.7 GeneMANIA, GSEA analysis and prediction of potential target drugs

The GeneMANIA database [39] is used to build networks. The database produces gene function hypotheses, analyzes gene lists, and establishes gene priority using functional analysis. Functionally linked genes may be found and weighted following expected values based on whole genome and proteome data to create core gene networks. Forecast potential targeted therapies for VaD using the DSigDB database (http://tanlab.ucdenver.edu/DSigDB). DSigDB database collects drug- and small molecule-related gene sets based on quantitative inhibition and drug-induced gene expression change data. We imported the obtained hub genes into the DSigDB database and used P < 0.05, Combined score >2000 as the conditions for screening target drugs [40].

### 2.8 Construction of ceRNA networks for hub genes

To start, we predicted the miRNAs for the four Hub genes using the intersection of Starbase [41], miRTarBase [42], and TargetScan database [43]. The co-expression of miRNA and lncRNA was researched using the intersecting miRNAs and Starbase. The relevant miRNAs and lncRNA were selected to form ceRNA networks based on the findings of the co-expression research, and the outcomes were shown using Cytoscape.

### 2.9 Immune infiltration analysis

CIBERSORT, a computational approach employing tissue gene expression patterns, was utilized to determine the proportion of immune cells in VaD and the control group [44]. This method estimated the number of immune cells in samples. We utilized box plots to display the resistant cell composition of patients with diverse immunological patterns. We computed 22 resistant cell types in patients using the CIBERSORT method from the R package [45]. The Pearson test was used to examine variations in immune cell ratios. *P* < 0.05 was statistically vital. The immunological score of recognizable immune cells and the amount of expression of diagnostic marker genes were analyzed using the Pearson correlation test. The significance of genes linked to VaD in connection to immune cells was determined using Pearson correlation analysis [46].

### 2.10 Statistical analysis

SPSS version 27.0 (IBM Corporation, Armonk, NY, USA) was used to construct ROC curves to establish and calculate AUC and 95% CI. The ROC curves were analyzed by GraphPad Prism(Version 9.5.1， San Diego, CA, USA). P < 0.05 was considered statistically significant.

## 3 Results

### 3.1 Data processing and screening of differentially expressed genes

**Fig. 1:**
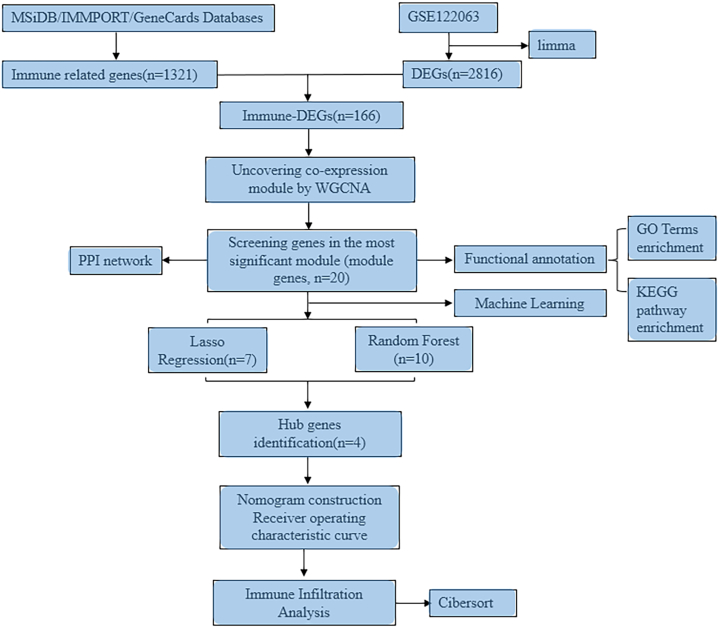
The flow chart of the study.

**Fig. 2:**
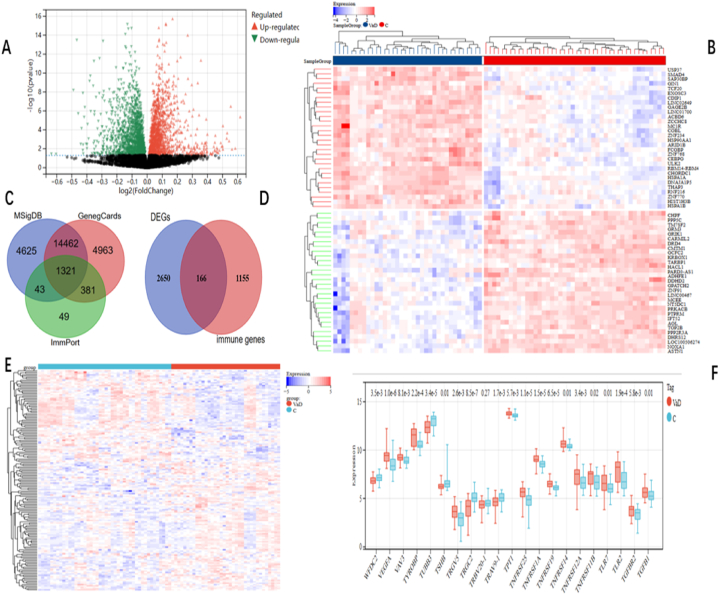
**Immune-related genes are differently expressed** (A) Green nodes represent downregulated differentially expressed genes (DEGs), red nodes represent upregulated DEGs, and black nodes represent genes that don't seem significantly differentially expressed. (B) Control samples are shown in scarlet in the heat map of VaD-related DEG expression levels, whereas ill samples are highlighted in navy blue. (C) Immune genes Venn's diagram (D) Immune genes and DEGs Venn's diagram (E) Heat map of immune genes and DEGs expression levels (F) Bar graph showing the expression level of immune cells concerning DEGs: green for healthy samples, red for unhealthy samples. (For interpretation of the references to color in this figure legend, the reader is referred to the Web version of this article.)

Fig. 1 depicts the flow chart for this study's bioinformatics analysis. Information sets were obtained from the GEO database, including brain sample information from 36 VaD patients and 44 controls. In a comparison of the VaD and control samples, 2816 DEGs were found, of which 1277 exhibited upregulation, and 1539 indicated downregulation (Fig. 2A and B). In Fig. 2C, three immune datasets' immune-related genes interacted with the DEGs to create 166 immune DEGs (Fig. 2D). Fig. 2E and F shows the heat map and bar graph for the 166 immune-DEGs.

### 3.2 Analysis and correlation of WGCNA module recognition

**Fig. 3:**
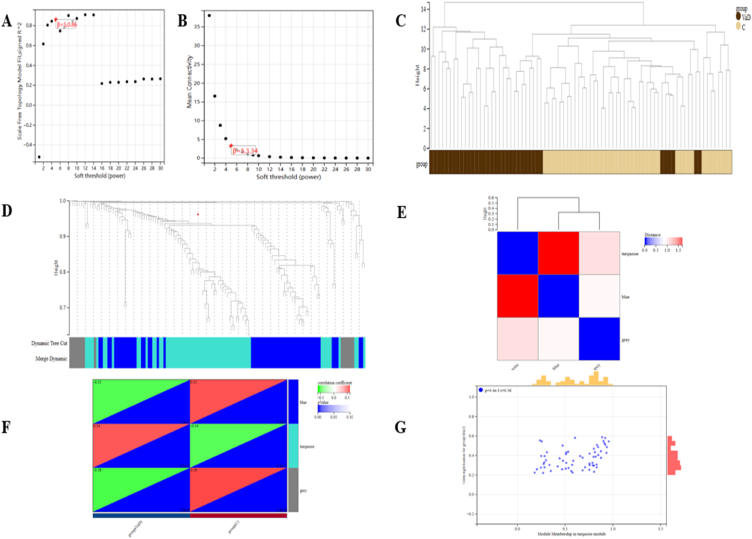
**Genes associated with DEGs module identification in VaD through WGCNA.** (A, B) β = 5 is chosen as the soft threshold due to the combined investigation of scale independence and average property. Agglomeration dendrogram of the control and VaD samples (C) the VaD and control sample aggregation dendrogram. (D) Completely distinct colors indicate factor co-expression modules. (E) Heatmap of eigengene contiguousness (F) Heatmap showing how modules and VaD are related. It is demonstrated that the turquoise module significantly correlates with VaD. (G) Correlation plot between gene significance and module membership. (For interpretation of the references to color in this figure legend, the reader is referred to the Web version of this article.)

WGCNA was used to identify VaD's primary correlative module. We preferred to select β = 5 (scale-free R^2^ = 0.86) because the "soft" threshold supported the dimensions independent and average characteristics (Fig. 3A and B). Fig. 3C depicts the cluster dendrogram of the VaD and control. Three co-expression modules (GCMs) were created using this power and are displayed in Fig. 3D and E in various hues. Fig. 3F depicts the association between VaD and GCMs, with the turquoise module (n = 20 genes) having, without a doubt, the best correlation with VaD (correlation constant = 0.54, p = 2.1 * 10^7^) and is considered crucial for further investigation. We calculate the associations between module membership and gene importance in the VaD turquoise module, and They were found to be correlated statistically (r = 0.36), as demonstrated in Fig. 3G.

### 3.3 Functional enrichment analysis and PPI construction

**Fig. 4:**
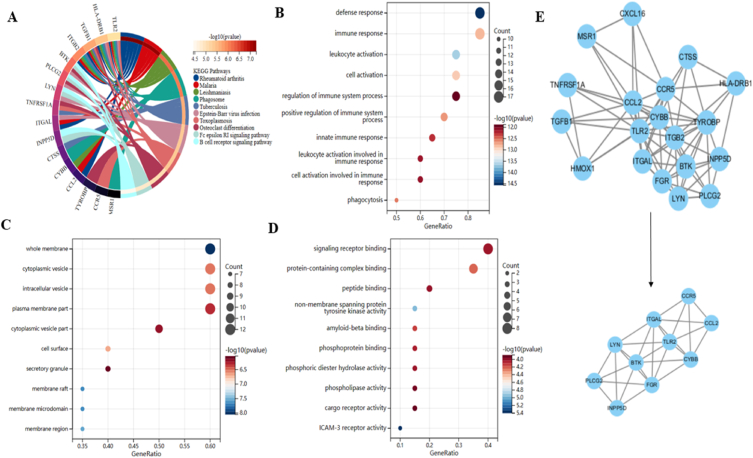
**Enrichment analysis of common genes from VaD.** (A) Gene intersection analysis using the KEGG pathway Many critical pathways and various distinct colors represent associated enriched genes. (B–D) GO analysis of the genes interacting with different biological processes, cellular components, and molecular functions. (E) Twenty genes interact, according to the PPI network, and the MCODE plug-in is used to depict the most important module. (For interpretation of the references to color in this figure legend, the reader is referred to the Web version of this article.)

According to KEGG analysis, "rheumatoid arthritis" and "malaria" were the two conditions where common genes (CGs) were most highly enriched (Fig. 4A). GO analysis showed that CGs were primarily improved in biological process (BP) terms, including "defense response" and "immune response" (Fig. 4B). Concerning cellular component (CC) metaphysics, the CGs primarily settled within the "whole membrane," "cytoplasmic vesicle," and "intracellular vesicle" (Fig. 4C). Molecular function (MF) analysis revealed that "signaling receptor binding," "protein-containing advanced binding," and "peptide binding" were the foremost vital things in the metric system (Fig. 4D). The enrichment analysis revealed that the VaD was predominantly linked to inflammatory responses, which were highly correlated with the pathologic process of VaD (Table 1).

**Table 1:** Functional analysis of intersection gene enrichment.

Term	Count	Adj. P value	Genes
**Biological Process**			
regulation of the immune system process	16	6.02E-12	TYROBP/TLR2/TGFB1/PLCG2/LYN/ITGB2/ITGAL/INPP5D/HMOX1/HLA-DRB1/FGR/EBI3/CTSS/CCL2/BTK
defense response	17	2.36E-11	TYROBP/TNFRSF1A/TLR2/TGFB1/PLCG2/LYN/ITGB2/ITGAL/HMOX1/HLA-DRB1/FGR/CYBB/CXCL16/CTSS/CCR5/CCL2/BTK
leukocyte activation	15	9.11E-11	TYROBP/TLR2/TGFB1/PLCG2/LYN/ITGB2/ITGAL/INPP5D/HMOX1/FGR/EBI3/CYBB/CTSS/CCL2/BTK
cell activation	15	9.11E-11	TYROBP/TLR2/TGFB1/PLCG2/LYN/ITGB2/ITGAL/INPP5D/HMOX1/FGR/EBI3/CYBB/CTSS/CCL2/BTK
immune response	17	1.03E-10	TYROBP/TLR2/TGFB1/PLCG2/LYN/ITGB2/ITGAL/INPP5D/HMOX1/HLA-DRB1/FGR/EBI3/CYBB/CXCL16/CTSS/CCL2/BTK
**Cellular Component**			
whole membrane	12	1.20E-06	TYROBP/TNFRSF1A/TLR2/MSR1/LYN/ITGB2/ITGAL/INPP5D/HMOX1/HLA-DRB1/CYBB/BTK
membrane raft	7	1.20E-06	TNFRSF1A/TLR2/LYN/ITGB2/INPP5D/HMOX1/BTK
membrane microdomain	7	1.20E-06	TNFRSF1A/TLR2/LYN/ITGB2/INPP5D/HMOX1/BTK
membrane region	7	1.20E-06	TNFRSF1A/TLR2/LYN/ITGB2/INPP5D/HMOX1/BTK
cell surface	8	7.87E-06	TYROBP/TNFRSF1A/TLR2/TGFB1/ITGB2/ITGAL/HLA-DRB1/CCR5
**Molecular Function**			
ICAM-3 receptor activity	2	0.00069	ITGB2/ITGAL
Non-membrane spanning protein tyrosine kinase activity	3	0.00093	LYN/FGR/BTK
protein-containing complex binding	7	0.0022	TLR2/MSR1/LYN/ITGAL/HLA-DRB1/FGR/CTSS
amyloid-beta binding	3	0.0022	TLR2/MSR1/ITGB2
phosphoprotein binding	3	0.0022	PLCG2/LYN/FGR
**KEGG Pathway**			
Rheumatoid arthritis	6	4.62E-06	TLR2/TGFB1/ITGB2/ITGAL/HLA-DRB1/CCL2
Malaria	5	4.62E-06	TLR2/TGFB1/ITGB2/ITGAL/CCL2
Leishmaniasis	5	2.87E-05	TLR2/TGFB1/ITGB2/HLA-DRB1/CYBB
Phagosome	6	2.89E-05	TLR2/MSR1/ITGB2/HLA-DRB1/CYBB/CTSS
Tuberculosis	6	6.02E-05	TNFRSF1A/TLR2/TGFB1/ITGB2/HLA-DRB1/CTSS

We built a PPI network to hunt for node genes that would collaborate during the ensuing machine-learning filtering once it was demonstrated that the screened genes were extremely strongly connected to immunity. The PPI network with 20 genes that may interact with one another is shown in Fig. 4E; hub genes are shown using the MCODE plug-in.

### 3.4 Finding potential hub genes using machine learning

**Fig. 5:**
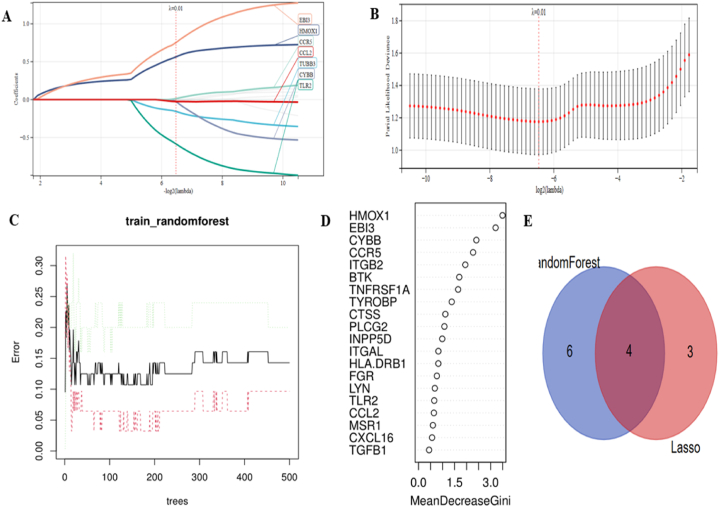
**Evaluating potential diagnostic biomarkers for VaD using machine learning.** (A, B) The Lasso model's biomarker screening. (C, D) The random forest algorithmic program displays the error, and the hierarchical management cluster and genes support the critical score. (E) The Venn figure illustrates two methods used to identify four candidate diagnostic genes.

Candidate genes were screened using LASSO regression and RF machine learning techniques for nomograph creation and diagnostic price analysis. Fig. 5A and B shows that the LASSO regression algorithmic program identified seven probable candidate biomarkers and that the RF algorithmic program hierarchically organized the sequences to support the estimation of each gene's relative relevance (Fig. 5C and D). When the Venn diagram containing the intersection of the top 10 most crucial genes from the RF and the seven likely candidate genes from LASSO was displayed (Fig. 5E), four genes (HMOX1, EBI3, CYBB, and CCR5) were already known for final confirmation.

### 3.5 Validation of VaD differential genes

**Fig. 6:**
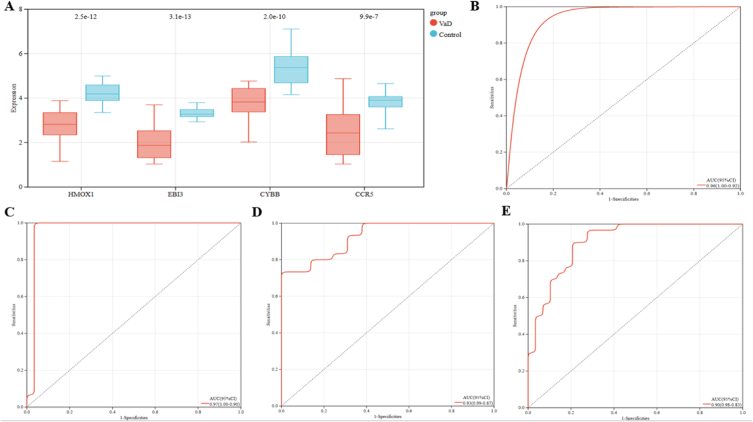
(A) Box plot showing the expression of target genes. （B-E）ROC curves for HMOX1, EBI3, CYBB, and CCR5.

To validate the reliability of the target genes, we used the GSE186798 dataset from the UK. Box plots were used to show the screened target genes' expression results in the independent dataset. The results showed that the expression of HMOX1, EBI3, CYBB, and CCR5 significantly differed in the validation set compared to the test set GSE122063 (Fig. 6A). The validation set also exhibited high confidence in the four Hub genes, HMOX1 (AUC 0.96, CI 0.92–1.00), EBI3 (AUC 0.97, CI 0.90–1.00), CYBB (AUC 0.93, CI 0.87–0.99), and CCR5 (AUC 0.90, CI 0.83–0.98), according to the charting of ROC curves (Fig. 6B–E).

### 3.6 Diagnostic value Assessment

**Fig. 7:**
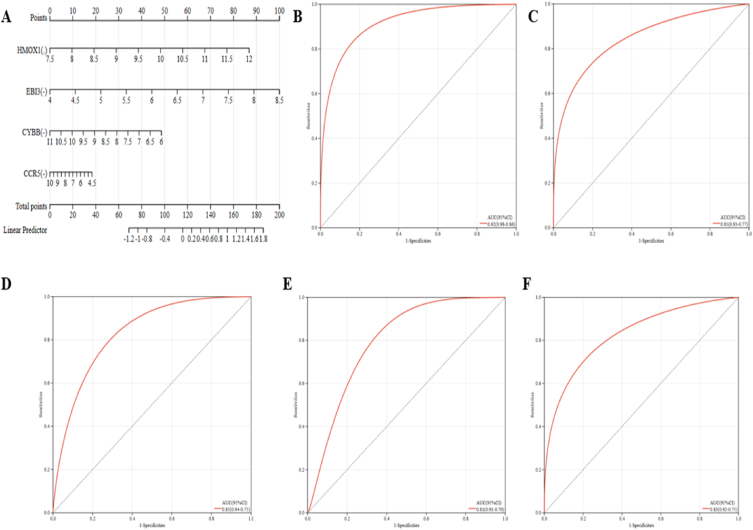
**Nomogram construction and analysis.** (A) The visible representation for VaD. (B–F) The ROC curve of the nomogram and every candidate factor (HMOX1, EBI3, CYBB, and CCR5).

A nomograph was built to support the four probable hub genes (Fig. 7A), and a ROC curve was created to evaluate the specificity and sensitivity of each component for diagnosing disease and the nomograph itself. For each item, we typically calculate the AUC and 95% CI. Nomogram (AUC 0.92, CI 0.86–0.98) was the highest value, followed by HMOX1 (AUC 0.85, CI 0.77–0.93), EBI3 (AUC 0.85, CI 0.75–0.94), CYBB (AUC 0.81, CI 0.70–0.91), and CCR5 (AUC 0.83, CI 0.75–0.92) (Fig. 7B–F). While all potential genes have potent diagnostic values for VaD, the nomogram has a maximum level of diagnostic value.

### 3.7 GeneMANIA, GSEA enrichment analysis, and drug prediction for four hub genes

**Fig. 8:**
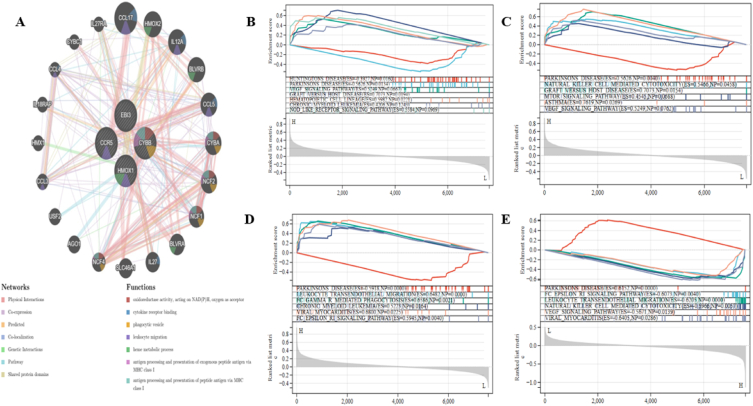
（A）PPI network construction.（B-E）GSEA analysis of HMOX1, EBI3, CYBB, and CCR5.

Fig. 8A shows the PPI network with HMOX1, EBI3, CYBB, and CCR5 activities. The GeneMANIA investigation revealed that these pathways are closely connected to the immunological inflammatory response. Then, we used GSEA to look into four Hub genes. These genes interact with leukocytes, NK natural killer cells, and Parkinson's disease pathways. These results are consistent with the significant VaD pathogenesis pathways and suggest that the four Hub genes are associated with immunological, inflammatory, and CNS degeneration (Fig. 8B–E). The Enrichr database (https://maayanlab.cloud/Enrichr/) was utilized to conduct our final drug discovery search targeting the four Hub genes of VaD. The top 15 medications in the DSigDB database that may affect the expression of the 4 Hub genes are displayed in Table 2.

**Table 2:** Potential top drug Compounds for VaD.

Term	Adj p-value	Combined score
Thiamet G	0.02732	3174
Palonosetron	0.02751	3172.3
SH-4-54	0.02771	3170.8
HX 531	0.02771	3170.8
ZM 241385	0.02771	3170.8
Betamethasone	0.02791	2169.2
Paliperidone	0.02791	2169.2
Atosiban	0.02791	2169.2
Tasimelteon	0.02791	2169.2
Ivacaftor	0.02791	2169.2
ABT-737	0.02791	2169.2
Forodesine	0.02791	2169.2
Alectinib	0.0281	2167.7
PD 160170	0.0281	2167.7
ML221	0.0281	2167.7

### 3.8 Results of ceRNA network construction

**Fig. 9:**
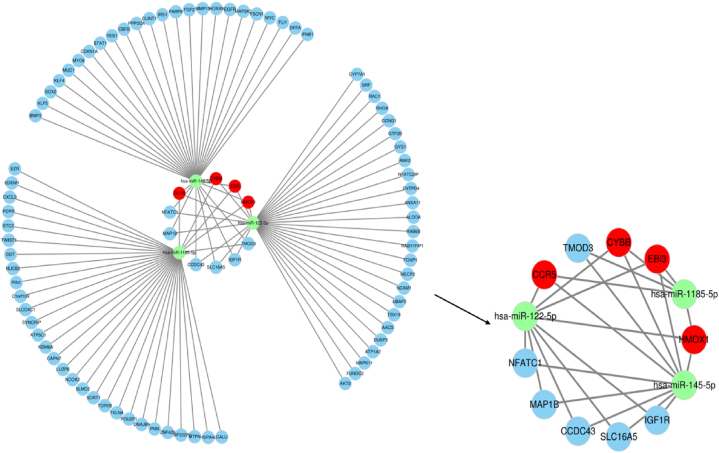
**The integrated ceRNA network.** Red circle: hub gene. Blue circle: lncRNA. Green circle: miRNA. (For interpretation of the references to color in this figure legend, the reader is referred to the Web version of this article.)

Different mechanisms exist for miRNA and lncRNA to regulate gene expression. Through the miRNA response region in ceRNA, lncRNA competitively binds to miRNA, reducing gene silencing by separating miRNA from mRNA [47]. The three data sets' intersection produced. Hub miRNAs miR-145-5p, miR-122-5p, and miR-1185-5p were used in the investigation's additional analysis. The ceRNA network was constructed using three miRNAs and four hub genes (HMOX1, EBI3, CYBB, and CCR5). We decided to add lncRNA in our analysis since it frequently appears in miRNA prediction findings. The results showed that the three miRNAs interacted with the four Hub genes through various lncRNA, including interactions with IGF1R, SLC16A5, CCDC43, MAP1B, NFATC1, and TMOD3 (Fig. 9). To expand our search for a regulatory mechanism for vascular dementia, we chose two miRNAs (miR-145-5p and miR-122-5p) and one lncRNA (IGF1R) published in research. We boldly assume that CYBB/miR-145-5p/IGF1R and CYBB/miR-122-5p/IGF1R may be exploited as novel regulators of VaD pathogenesis.

### 3.9 Immune cell infiltration analysis

**Fig. 10:**
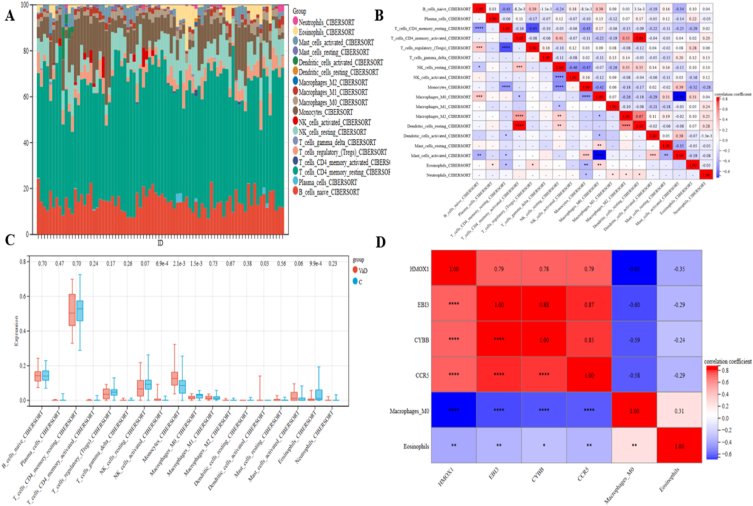
**Investigation of immune cell infiltration in the VaD and control groups.** (A) the percentage of 18 different resistant cell types present in diverse samples that match the barplot's predictions (B) Correlation of the cell types of 18 immune cells (C) 18 categories of immune cells' proportions in the VaD and control teams were compared (D) Correlation between immune cells and four diagnostic biomarkers.

The correlation of 18 teams of immune cells within the blood of VaD patients was first evaluated. Because memory B cells, CD8^＋^T cells, naive CD4^＋^T cells, and T follicular helper immune cells had no expression within the sample, we tended to remove them within the next step of the analysis (Fig. 10A). In line with the results, activated CD4^＋^T memory cells and resting dendritic cells had a significant positive correlation. Macrophages M0 cells and activated mast cells had a significant negative correlation. The Pearson test was used to evaluate substantial variations in immune cell infiltration in peripheral blood between VaD patients and, therefore, the control group (Fig. 10B). We are more calculable the proportion of 18 immune cell subtypes. Fig. 10C displayed the immune cells' dispersion, and activated NK cells, monocyte cells, Macrophages M0 cells, activated dendritic cells, and eosinophil cells were differentially distributed. The results show completely different immune cell occupancy within the peripheral blood of VaD patients and controls. Macrophages M0 cells and Eosinophils cells were considerably reduced in VaD patients compared to controls, confirming the importance of reducing this kind of cell within the VaD immune microenvironment.

The correlation between four valid biomarkers (HMOX1, EBI3, CYBB, and CCR5) and two considerably different immune cells (Macrophages M0 cells, Eosinophils cells) is shown in Fig. 10D. Four validated biomarkers showed a significant negative correlation with macrophage M0 cells and a negative correlation with eosinophil cells. Individuals with VaD often had different immune cell subsets infiltrate to varying degrees, which may be a regulatory target for VaD treatment.

## 4 Discussion

High-throughput methods and microarray technology are valuable tools for enhancing our comprehension of the fundamental molecular processes underlying advanced illnesses. Vascular dementia, the second most common kind of dementia after Alzheimer's, has a considerable impact on patients' everyday lives. Despite this, the molecular and cellular processes underlying the pathobiology of VaD have yet to be well understood. This research gap is mainly caused by the diversity of sickness in clinical settings and the need for more sufficient experimental models to faithfully reproduce most pathogenic pathways underlying the genesis and development of different kinds of VaD. It is currently understood that the result of brain damage brought on by neurovascular injury over time plays an integral part in the pathobiology of VaD [[48], [49], [50]]. Therefore, the hunt for new targets for the first identification, treatment, and prognosis of the immune microenvironment in vascular dementia is of great clinical importance.

All vascular dementia and control samples during this study contained 2816 differentially expressed sequences. One hundred sixty-six were immune-related genes that overlapped with the immune gene information. We ran GO and KEGG pathway enrichment studies to learn more about how DEGs and their signaling pathways work. According to our research, these DEGs were predominantly abundant in immunological responses, inflammatory responses, leukocyte activation, positive immune system process regulation, signal receptor binding, and immune-mediated signal pathways. There is growing proof that inflammatory and immune responses play a crucial role in the development of VaD and associated noninheritable intellectual incapacity syndrome caused by vessel sickness, within which risk factors for vessel sickness are concerned within the pathologic process. Therefore, the factors influencing the vessel pathologic process are the most crucial reason behind VaD. The chance factors for vessel sickness are concerned with its pathologic process, and therefore, the factors influencing the vessel pathologic process are also the most common causes of VaD. One study [51] showed that T cells and post-ischemic cytokines are concerned with cerebral anemia injury, and immune and inflammatory responses are the most important factors resulting in secondary injury in cerebral anemia. Additionally, machine learning methods and the construction of nomograms haven't been used for vascular dementia. Here, utilizing various integrated bioinformatics analysis and machine learning approaches, we constructed nomograms and assessed their diagnostic usefulness for vascular dementia. We are aware of 4 crucial immune-related candidate genes (HMOX1, EBI3, CYBB, and CCR5). Heme oxygenase-1 (Hmox1), a stress-inducible enzyme, catalyzes hemoglobin's disintegration into iron, monoxide, and hemosiderin. Both disease and anemia harm are protected against by inducing Hmox1 and its metabolites. Hmox1 breaks down hemoglobin into ferrous iron, bilirubin, and carbon monoxide, which are swiftly converted to hemoglobin by the hemoglobin enzyme. Hmox1 is an inducible isoform of the enzyme hominin oxidoreductase, and various stressors, including hemoglobin, aerophilic stress, UV radiation exposure, and drive, can induce its transcription. Inducing Hmox1 is a crucial part of the vascular system's response to damage and healing in diagnostic models [52]. For instance, sequence therapy-mediated upregulation of Hmox1 suppresses endothelial hyperplasia and vascular smooth muscle cell proliferation [53] and promotes epithelial cell growth [54] and epithelial reformation [55]. It also provides semi-permanent protection against cerebral ischemia/reperfusion injury [56]. The complex cytokines IL-27 and IL-35 comprise a component called Epstein-Barr virus-induced gene 3 (EBI3). Every protein has useful functions or effects in inflammatory and immune disease models. As proteins that have been known in recent years to play a vital role in immune regulation, they need to, step by step, become a hot topic in immune tolerance analysis [57]. Studies [58] have found the involvement of IL-6 in both pathological and physiological changes in VD, which has been tested considerably in healthy populations. IL-6 may be a protein that chiefly regulates the body's immune system and participates in the associated inflammatory response, mediating the onset of inflammatory reactions following external infections [59]. Among the various biomarkers of inflammation, IL-6 plays an outstanding role in identifying VaD. Studies have demonstrated that inflammatory cytokines, chemokines, adhesion molecules, and alternative pro-inflammatory mediators are overexpressed in the epithelium and smooth muscle cells as people age, creating a pro-inflammatory microenvironment that encourages vascular dysfunction [60]. EBI3 and IL-6 induce chemokine expression in human blood vessel epithelium cells. Studies have shown that EBI3 will promote pro-inflammatory IL-6 by mediating counter-signaling [57]. However, the precise mechanism of action of EBI3 in VaD needs additional confirmation. Cytochrome *b*-245 beta chain (CYBB) makes up hemoprotein b-245, a vital supermolecule of vegetative cell NADPH oxidase (NOX), a membrane-bound protein that, once activated, produces giant amounts of bactericidal superoxide and other oxides. There is growing proof that arteriosclerosis lesions of the vasculature are an indicator of VaD development [61], and oxidative stress (OS) plays an infective role in coronary artery disease and alternative vessel diseases [62]. Several risk factors for vas malady and tube dementia are related to elevated OS [63]. Associate degree imbalance within the quantitative relation of OS antioxidants to reactive oxygen species (ROS) harms tube epithelium cells, interstitial tissue cells, and neural cells, significantly decreasing cerebral blood flow and neurovascular uncoupling [64]. However, NOX is a severe source of ROS throughout aging, hypoperfusion, stroke, and cardiovascular disease [65]. NOX-generated ROS could be an effective ROS-producing system and is often related to the accrued OS ascertained in VaD and its risk factors [66]. The supermolecule C–C chemokine receptor type 5 (CCR5), found on the leukocyte surface, is encoded by the CCR5 sequence and functions in the body's chemokine receptor system to help T lymphocytes adhere to particular tissues and target organs. It controls T-cell migration, white or scavenger cell lineage growth, and immunity. It is mainly expressed on the membranes of immature nerve fiber cells, monocytes, and memory-resting T lymphocytes [67]. In previous studies [68], CCR5 was known as a hub gene for VaD. However, no specific mechanism of action was elaborated.

Following discovering possible Hub genes for VaD, we predicted the miRNAs and lncRNAs linked to them using internet databases and built ceRNA networks to connect them. The Hub gene (CYBB), lncRNA (IGF1R), and miR-145-5p and miR-122-5p were the final areas of investigation. How oxidative stress induces apoptosis in neurodegenerative illnesses is a topic that researchers are increasingly interested in. VaD's pathogenesis has previously been linked to miR-145-5p and miR-122-5p [69], and miR-145-5p is strongly related to oxidative stress in neurodegenerative illnesses. IGF-1 (insulin-like growth factor-1) is implicated in the etiology of dementia, protects neurons by lowering their sensitivity to oxidative stress, and is strongly correlated with the A allele of the IGF1R polymorphism for VAD (60). We postulate that the ceRNA interaction network of CYBB/miR-145-5p/IGF1R and CYBB/miR-122-5p/IGF1R's role is crucial in the beginning and development of VaD through oxidative stress, which may give suggestions for treating VaD. Literature investigations and bioinformatics prediction analysis developed this hypothesis.

According to one study [70], recombinant lymphocyte immunity starts functioning in the immunological environment as people age. The pathophysiology of VaD is also significantly influenced by the immune system. The current study measures the degree of immune cell infiltration and looks at the pattern of immune cell infiltration in disease using the CIBERSORT method. We concentrate on distinctive diagnostic indicators and investigate how immune cell invasion affects VaD. As per previous findings, the results showed a considerably higher rate of NK cell and monocyte infiltration in VaD [71]. Innate immune cells include NK, monocytes, and neuroglia; the adaptive immune system comprises B and T lymphocytes. Undeniably, as people age, their immune systems become less capable of controlling inflammation. The pathogenesis of dementia requires this ongoing inflammation of the brain's borders and tissues [72]. According to one study [73], The frequency of monocytes, NK cells, B cells, and memory T cells varied in peripheral blood from AD and VaD patients. These alterations, which result in the generation of cytokines and chemokines, are presumably the result of a turbulent interaction between peripheral and central immunity. These cell populations may have a significant impact on the onset of dementia. Additionally, macrophages and eosinophils were considerably downregulated in VaD patients compared to controls. All four known immune hub genes were negatively correlated with these two cells, suggesting that a reduction within these two cells is vital in the VaD immune microenvironment.

Biomarkers are quantifications of well-defined biological states that are usually related to the danger, incidence, severity, prognosis, or expected response to treatment of a disease. The discovery of biomarkers will guide scientists' efforts to create early-detectable peripheral biomarkers, enabling us to comprehend the causes and processes of certain conditions. Single biomarkers might not be adequate to spot the potential complexity of cellular changes related to a disorder, and biomarkers reflecting completely different pathophysiological options of infection or syndrome could also be needed to capture the complexity of a selected condition [74]. There has been a hunt for biomarkers to boost the diagnosis of stroke, vascular dementia, and their etiology, and to realize this goal, we tend to screen and validate immune-related hub genes for VaD using bioinformatics strategies and perform immune cell infiltration analysis using CIBERSORT, and these results, particularly those related to immune cells, might provide new perspectives for diagnosing VaD. However, we recognize that this study also has certain limitations. First, due to differences in analytical thought and approach, our investigation may be a secondary mining of a previously released dataset and provide different findings. Second, the distribution of several low-abundance expressed immune cell subpopulations in VaD has yet to be wholly identified since the CIBERSORT algorithmic method only supports limited transcriptome data. Therefore, not only do our results support some earlier research, but they also cast doubt on some of it. Additional laboratory testing is required.

## 5 Conclusion

Using bioinformatic techniques, our research identified four immune-related candidate core genes (HMOX1, EBI3, CYBB, and CCR5). We created a network of hub genes and discovered potential therapeutic agents. In addition, based on literature analysis and bioinformatic predictions, we propose CYBB/miR-145-5p/IGF1R and CYBB/miR-122-5p/IGF1R as prospective RNA regulatory pathways that may control the progression of VaD. Our work clarifies significant immune-infiltrating expression in the field by identifying critical genes in VaD bioinformatics and delving into their underlying mechanisms. It advances our understanding of the molecular mechanisms underlying vascular dementia, which may lead to novel therapeutic approaches.

## Ethics approval and consent to participate

The raw data of this study comes from public databases and does not require ethical approval.

## Consent for publication

Not applicable.

## Funding

The present study was funded by the 10.13039/501100007129Natural Science Foundation of Shandong Province (ZR2022MH124), the Youth Science Foundation of 10.13039/501100015507Shandong First Medical University (202201-105), the Shandong Medical and Health 10.13039/100006180Technology Development Fund (202103070325), the Shandong Province Traditional Chinese Medicine Science and Technology Project (M − 2022216).

## Availability of data and materials

Publicly available datasets were analyzed in this study. This data can be found at https://www.ncbi.nlm.nih.gov/geo/query/acc.cgi?acc=GSE122063,and https://www.ncbi.nlm.nih.gov/geo/query/acc.cgi?acc=GSE186798. All data generated or analyzed during this study are included in this published article.

## CRediT authorship contribution statement

**Fangchao Wu:** Writing – original draft. **Junling Zhang:** Data curation, Conceptualization. **Qian Wang:** Formal analysis, Data curation. **Wenxin Liu:** Methodology, Investigation. **Xinlei Zhang:** Resources, Project administration. **Fangli Ning:** Resources, Project administration. **Mengmeng Cui:** Supervision, Conceptualization. **Lei Qin:** Resources. **Guohua Zhao:** Software. **Di Liu:** Validation. **Shi Lv:** Writing – review & editing. **Yuzhen Xu:** Writing – review & editing, Funding acquisition.

## Declaration of competing interest

The authors declare that they have no known competing financial interests or personal relationships that could have appeared to influence the work reported in this paper.
